# Nontraumatic spontaneous intracerebral hemorrhage: Baseline characteristics and early outcomes

**DOI:** 10.1002/brb3.1512

**Published:** 2019-12-15

**Authors:** Mohamed Al‐Khaled, Samer Awwad, Toralf Brüning

**Affiliations:** ^1^ Department of Neurology University of Lübeck Lübeck Germany

**Keywords:** hemorrhage, nontraumatic spontaneous intracerebral hemorrhage, prognosis

## Abstract

**Background and Purpose:**

Hemorrhagic stroke, particularly nontraumatic spontaneous intracerebral hemorrhage (SICH), is a cerebrovascular condition with unfavorable outcomes. The aims of the present study were to evaluate patients who suffered from SICH and investigate the early outcomes in a single‐center study.

**Methods:**

During a study ‐period of 6 years (2008–2014), 613 consecutive patients (mean age, 72 ± 12.7 years; 51.1% female), who suffered from nontraumatic SICH and were treated at the Department of Neurology at the University Hospital of Schleswig‐Holstein, Campus Lübeck, Germany, were included and prospectively analyzed.

**Results:**

During a mean hospitalization time of 12 days, 148 patients (24.1%) died, 47% of those within the first 2 days and 79% within the first week. The patients who died stayed at the hospital for a shorter time (3) than those who survived (*p *< .001). In the multivariate logistic regression, following parameters were found to be associated with the in‐hospital mortality: female sex (OR, 2.0; 95%‐CI, 1.2–3.4; *p* = .009), a NIHSS score> 10 (OR, 10.5; 95%‐CI, 5.6–19.5; *p *< .001), history of hypertension (OR, 0.35; 95%‐CI, 0.19–0.64; *p *= .001), previous oral anticoagulation (OR, 2; 95%‐CI, 1.0–3.8; *p *= .032), and intraventricular extension of hemorrhage (OR, 2.8; 95%‐CI, 1.7–4.7; *p *= .001). At discharge, 192 patients (41.2%) showed favorable outcomes (mRS ≤ 2) whereas the median mRS of patients who survived was 3 (IQR 2–4). The good functional outcome at discharge from the acute hospital was decreased by an age> 70 years (OR, 0.56; 95%‐CI, 0.35–0.9; *p *= .017), NIHSS score> 10 at admission (OR, 0.07; 95%‐CI, 0.04–0.13; *p *< .001), and development of pneumonia during hospitalization (OR, 0.35; 95%‐CI, 0.2–0.6; *p *< .001).

**Conclusion:**

The present study showed that SICH is a serious disease causing high mortality and disability, particularly in the early period after event.

## Graphical abstract

This article gives an overview of the baseline characteristics, therapeutic procedures, and early outcomes in patients suffering from spontaneous intracerebral hemorrhage.

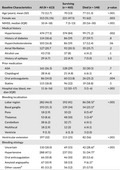

## 1 INTRODUCTION

The hemorrhagic stroke, nontraumatic spontaneous intracerebral hemorrhage (SICH), is a heterogeneous cerebrovascular condition that leads to disability and rapid death (Al‐Khaled, Eggers, & Qug, 2014; Broderick et al., 2007; Weimar et al., 2003). It represents the second form of stroke with a number of patients affected worldwide each year as high as 4 million and a median case fatality of one month of 40% (Asch et al., 2010; Ferro, 2006; Kolominsky‐Rabas et al., 1998; Sacco et al., 1998; Sadamasa, Yoshida, Narumi, & Chin, 2011). In such a way, SICH is characterized as the most killing disease in the early phase after onset (Hemphill, Farrant, & Neill, 2009). Many survivors remain severely disabled with only one in four patients having a good outcome (Feigin, Lawes, Bennett, & Anderson, 2003).

**Figure 1:**
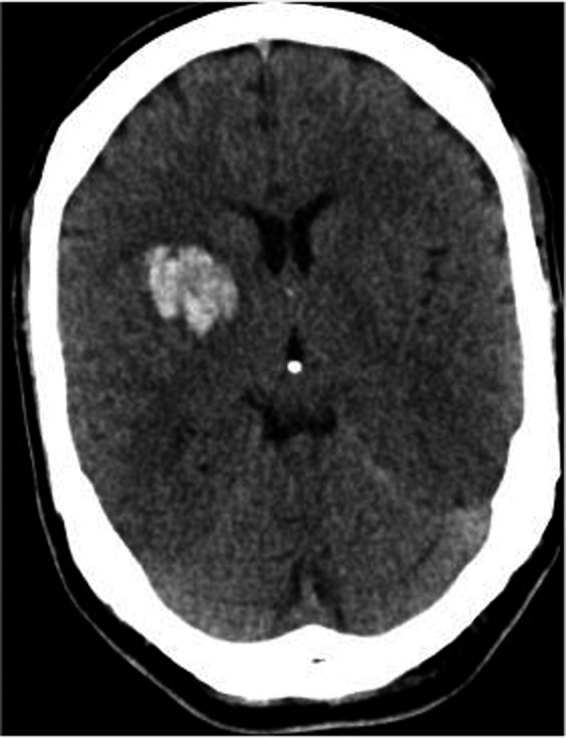
Brain CT scan showed a SICH by a 45‐year‐old patient, firstly diagnosed with blood hypertension. The patient was presented with a weakness on the left side and hypertensive crisis

**Figure 2:**
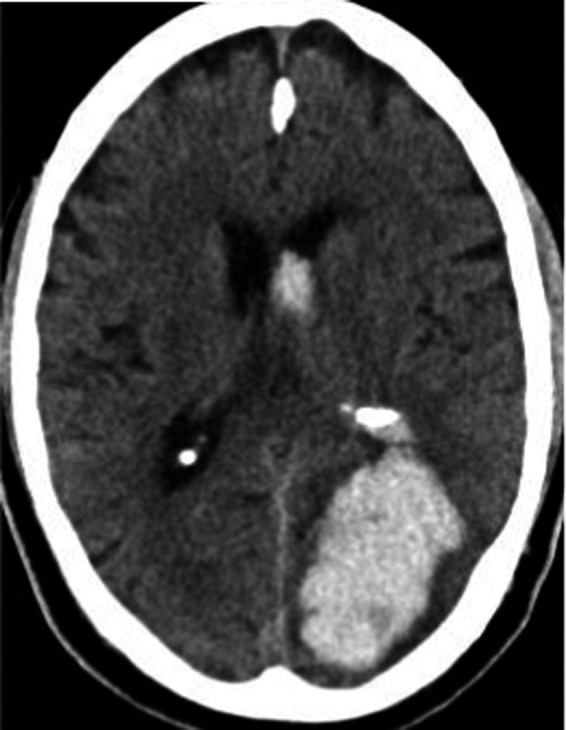
Atypically localized sICB with ventricular extension showed by brain CT scan. A 64‐year‐old woman who were presented with a fast‐progredient altered consciousness

**Figure 3:**
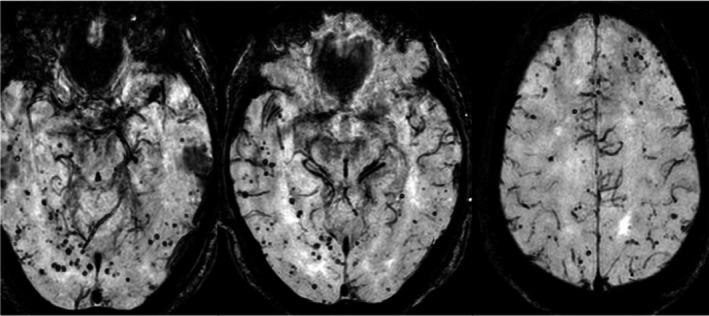
MRI scan showed cerebral amyloid angiopathy, several microbleeds in the infra‐ as well extratentorial region. A 67 years patients presented with increasing cognitive impairment in the last year

In SICH, the bleeding results from cerebrovascular vessels most likely due to first diagnosed or preexisting blood hypertension and as well as a result of amyloid angiopathy (Butcher and Laidlaw, 2003). The brain regions basal ganglia (34%) followed by lobar regions (25%) are the most frequently affected sites in SICH (Hemphill, Bonovich, Besmertis, Manley, and Johnston, 2001). The SICH occurs typically in the basal region by hypertension (Figure 1), whereas it may atypically occur in other brain regions (Figure 2). The cerebral amyloid angiopathy causes SICH as well as asymptomatic microbleeds in the brain that can be detected by brain MRI (Figure 3).

Whereas an absolute benefit from surgery could have been revealed recently in patients with traumatic intracerebral hemorrhage (Gregson et al., 2015), the potential benefit of surgical compared to conservative treatment of SICH still remains controversial, a significant minor advantage was found in cases of SICH without intraventricular extension (Kim, Lee, Koh, & Choi, 2015; Mendelow et al., 2013).

## 2 METHODS

### 2.1 Study design

Between 2008 and 2014, 613 patients (mean age, 72 ± 12.7 years; female, 51.1%), who were suffering from SICH and were treated at the University Hospital of Schleswig‐Holstein, Campus Lübeck, Germany, were included in the present prospective study. We used standardized radiological analysis in characterizing SICH.

The diagnosis of SICH was made by using cranial computed tomography (CCT) and magnetic resonance imaging of brain (cMRI).

Baseline parameters, diagnostic, and therapeutic procedures were retrieved from the medical patient's files (Table 1). The assessment of outcome was performed by the modified Rankin Scale (mRS), with a range from mRS = 0 points [no symptoms] to mRS = 6 points [death].

**Table 1:** Baseline characteristics and comparison between survival and death

Baseline Characteristics	All (*N* = 613)	Surviving (*n* = 465)	Died (*n* = 148)	*p*‐value
Age (years), mean (*SD*)	72 (12.7)	70 (13)	77 (11.3)	<.001
Female sex	313 (51.1%)	221 (47.5)	91 (62)	.003
NIHSS, median (IQR)	10 (4–18)	7 (3–13)	20 (16–30)	<.001
Medical history				
Hypertension	474 (77.3)	374 (84)	99 (71.2)	.002
History of diabetes	114 (18.6)	86 (19)	27 (19.7)	.8
Hypercholesterolemia	103 (16.8)	86 (19)	17 (12.4)	.06
Atrial fibrillation	127 (20.7	92 (20.5)	35 (25.7)	.2
Alcohol use	43 (7.0)	37 (8)	6 (4.2)	.1
History of epilepsy	29 (4.7)	22 (4.9)	7 (5.0)	1.0
Prior medication				
ASA	161 (26.3)	128 (29)	32 (30.5)	.7
Clopidogrel	28 (4.6)	21 (4.8)	6 (6.1)	.4
Oral anticoagulants	86 (14.0)	60 (13.8)	26 (25.2)	.004
Statins	115 (18.8)	96 (22)	19 (18.8)	.5
Hospital stay (days), median (IQR)	11 (6–16)	12 (10–17)	3 (1–6)	<.001
Bleeding localization				
Lobar region	282 (46.0)	192 (41)	86 (58.5)^a^	<.001
Basal ganglia	193 (31.5)	159 (34)	34 (23.1)^a^	
Pons	18 (2.9)	10 (2)	8 (5.4)^a^	
Thalamus	53 (8.6)	48 (10)	5 (3.4)^a^	
Cerebellum	38 (6.2)	32 (7)	6 (4.1)	
Multifocal	18 (2.9)	12 (3)	6 (4.1)	
Ventricle	9 (1.5)	6 (1.3)	3 (2.0)	
Ventricular extension	197 (32)	115 (25)	82 (56)	<.001
Bleeding etiology				
Uncertain	110 (18.0)	69 (15)	42 (28.6)^a^	<.001
Hypertensive	288 (47.1)	237 (51)	51 (34.7)^a^	
Oral anticoagulation	66 (10.8)	46 (10)	20 (13.6)	
Amyloid angiopathy	67 (10.9)	58 (13)	9 (6.1)^a^	
Other causes^b^	81 (13.2)	56 (12)	25 (17.0)	

Note

Values are in absolute number and percentage unless other indicated.

Abbreviations: ASA, acetylsalicylic acid; *SD*, standard deviation.

a Change's by Bonferroni‐corrections are significant.

b Indicates rare known causes (e.g., small vessel malformation, brain tumors, sinus venous thrombosis etc).

All deaths were assumed as neurological mortality. Favorable outcomes were set at a mRS ≤ 2. The patients were evaluated at the time of admission to the discharge from the hospital.

All included patients were admitted in the semi‐intensive care (stroke unit) or in the intensive care unit in cases of altered consciousness and/or when assistance of respirations was required. All patients were treated by physicians with stroke experience as well as stroke neurologists.

The present study was part of the Stroke Registry at the Department of Neurology. The study (Stroke Registry) was approved by the local ethics committee.

The patients, who were entered in this study, were exclusively treated conservatively. This conservative care was carried out in accordance with the recommendations and guidelines of the German Stroke Society and German Society of Neurology (European Stroke Initiative Writing C, 2006; Thieme Verlag, 2012). Briefly, the management of SICH was conservative and included controlling of blood pressure and other vital parameters, if necessary an intra‐arterial measurement was used. The monitoring of vital parameters was essential in the care of hemorrhage patients (Qureshi et al., 2010). Furthermore, patients with respiratory insufficiency due to altered consciousness received an intubation and ventilation for airway protection, those patients were treated initially on intensive care, after weaning the treatment was continued on the stroke unit.

### 2.2 Statistical analysis

The SPSS software (version 23; IBM) was used to analyze the data. The values of the continuous variables were reported with the mean and standard deviations, whereas the scores with the median and interquartile range (IQR). Absolute numbers and percentages were used to describe the categorical and nominal variables. A chi‐square test was used to determine the correlation between categorical variables and a Mann–Whitney test between scores as well as a *t* test between continuous variables was performed. The multivariate analysis was performed to estimate the odds ratio (ORs), and all parameters with a significant association in the univariate analysis were entered in the logistic regression. A *p*‐value below .05 was considered significant.

## 3 RESULTS

In the present study, a total of 613 patients (mean age 72 ± 12.7 year; 51.1% female; median NIHSS score 10 [IQR 4–18]) were included conservatively for nontraumatic SICH.

All patients have undergone at least one CCT scan, one at admission, and control CT scan during hospitalization or/and MRI scan.

During hospitalization with a mean overall duration of hospital stay of 12 ± 8 days, 148 patients (24.1%) died, 47% of those died within the first 2 days and 79% within the first week. The mean hospitalization of patients who did not survive was remarkably shorter (3 days) than that of those who survived (*p *< .001).

The baseline parameters and a comparison between patients who survived vs. those who did not are shown in Table 1. The rate of early death was found to be higher in the elderly, female gender, history of hypertension, and premedication with oral anticoagulants. Furthermore, a significant association between mortality and localization as well as etiology of bleeding has been established (Table 1).

In the multivariate logistic regression, following parameters were found to be associated with the in‐hospital mortality: female sex (OR, 2.0; 95%‐CI, 1.2–3.4; *p* = .009), a NIHSS score > 10 (OR, 10.5; 95%‐CI, 5.6–19.5; *p *< .001), previous oral anticoagulation (OR, 2; 95%‐CI, 1.0–3.8; *p *= .032), and intraventricular extension of hemorrhage (OR, 2.8; 95%‐CI, 1.7–4.7; *p *= .001) that were associated with increased death, contrarywise a known hypertension was linked to decreased in‐hospital mortality (OR, 0.35; 95%‐CI, 0.19–0.64; *p *= .001) in logistic regression analysis.

At discharge, 192 patients (41.2%) showed favorable outcomes (mRS ≤ 2) whereas the median mRS of patients who survived was 3 (IQR 2–4). Patients who showed favorable outcomes seem to be younger, less affected at admission, had hypercholesterolemia and had suffered the bleeding in the lobar region (Table 2).

**Table 2:** Baseline characteristics, risk factors, bleeding localization, and complications of patients with spontaneous intracerebral hemorrhage

Baseline characteristics	Functional outcome at discharge (*N* = 445)		*p*‐value
	Good outcome; mRS = ≤2 (*n* = 193)	Poor outcome; mRS = 3–5 (*n* = 252)	
Age (years), mean (*SD*)	68 (12)	72 (13)	.002
Female gender	87 (45.3%)	125 (50)	.3
NIHSS, median (IQR)	3 (2–6)	12 (7–16)	<.001
Medical history			
Arterial hypertension	156 (82)	213 (85)	.2
Alcohol abuse	11 (5.7)	25 (10)	.1
Diabetes mellitus	35 (18.3)	51 (20)	.5
Hypercholesterolemia	47 (24.6)	39 (15.6)	.019
Atrial fibrillation	34 (17.7)	57 (23)	.2
Prior medication			
ASS	58 (30.5)	68 (28)	.6
Clopidogrel	8 (4)	13 (5)	.5
Anticoagulants	26 (14)	34 (14)	.9
Statin	47 (25)	49 (20)	.2
Hospitalization length (days), median (IQR)	11 (8–13)	15 (11–20)	<.001
Bleeding localization			
Lobar	91 (47.4)	93 (37)^a^	.058
Basal ganglia	56 (29.2)	94 (37.5)	
Pons	5 (2.6)	5 (2.6)	
Thalamus	15 (7.8)	30 (12)	
Cerebellum	15 (7.8)	16 (6.4)	
Multifocal	2 (1.0)	10 (4)	
Ventricular	5 (2.6)	1 (0.4)	
Hypophysis	1 (0.5)	0	
Intraventricular extension	31 (16)	80 (32)	<.001
Bleeding etiology			
Uncertain	32 (16.7)	32 (12.7)	.8
Hypertensive	89 (46.4)	139 (55)	
Oral anticoagulation	21 (10.9)	25 (10)	
Amyloid angiopathy	25 (13.0)	29 (11.6)	
Other causes^b^	25 (13.0)	23 (9)	

Note

Values in numbers and percentage unless otherwise indicated.

Abbreviations: ASS, acetylsalicylic acid; IQR, interquartile range; *SD*, standard deviation.

a Indicates that changes in the Bonferroni methods are significant.

b Indicates rare known causes (e.g., small vessel malformation, brain tumors, sinus venous thrombosis etc).

In the multivariate logistic regression analysis, a good functional outcome at discharge from the acute hospital was decreased by an age >70 years (OR, 0.56; 95%‐CI, 0.35–0.9; *p *= .017), NIHSS score >10 at admission (OR, 0.07; 95%‐CI, 0.04–0.13; *p *< .001), and development of pneumonia during hospitalization (OR, 0.35; 95%‐CI, 0.2–0.6; *p *< .001).

## 4 DISCUSSION

Intracerebral hemorrhage is a cerebrovascular disease with poor outcomes. It is associated with a considerably high rate of disability and mortality, especially in the short time following the event, regardless of conservative management or interventional care (Kim et al., 2015; Mendelow et al., 2013; Qureshi et al., 2010; Qureshi & Qureshi, 2018; Trabert & Steiner, 2012).

Comparable to previous study results (Hemphill et al., 2001; Mayer & Rincon, 2005), the intracerebral hemorrhage seems to be very lethal in the early short period after onset. We noticed in the group that did not survive the hemorrhage that four out of five patients died within the first week. However, it is important to note that patients who died in the hospital were older and much more severely neurologically impaired by the time of admission to the hospital than those who survived. In Addition, the mortality was associated with gender, consciousness level, history of hypertension, and anticoagulation as well as the localization of bleeding. Similar findings have been shown in survival with good outcomes.

Conservative treatment focuses on regulating and controlling blood pressure and the treatment of hypertension. Reduction in systolic blood pressure to values between 130 and 150 mm Hg, particularly in the early phase of symptom onset could play a role in reducing the expansion of intracerebral bleeding (Qureshi et al., 2010; Qureshi & Qureshi, 2018).

Contrarywise, we found that the previous hypertension was associated with survive of bleeding in the logistic regression analysis. This could be related to the effectiveness of the preexisting hypertensive drugs that predict hypertensive blood pressure above 180mm Hg and bleeding expansion.

Recent study (Bernardo, Rebordao, Machado, Salgado, & Pinto, 2019) showed that in an age between 18 and 65 years, the hospital mortality was found remarkably lower (14.9%) than that in our study with mean age of 70 and 77 years among patients who died. However, the age is uncontrollable predictor for the prognosis compared to other factors, for example, pneumonia that leads to worsening of prognosis (Lindner et al., 2019).

Studies have shown that up to 20% of hemorrhage survivors return to independency (mRS ≤ 2) at 6 months, and these rates fairly have changed over the past two decades (Flaherty et al., 2006; Grysiewicz, Thomas, & Pandey, 2008). Whereas the prognosis is related to the size and localization of hemorrhage, for example, hemorrhage lacunar stroke that accounts for 7.4% of cerebral hemorrhages is associated with a more favorable prognosis as compared to the remaining group of intracerebral hemorrhages, usually with survival and deficit‐free at discharge from the hospital in 22.8% of cases (Arboix et al., 2002; Arboix, Carica‐Eroles, Massons, Oliveres, & Targa, 2000).

In our study, the functional good outcome was significantly decreased in patients who were older (>70 years) and severely affected at admission (NIHSS score > 10) as well as in cases of development of pneumonia during hospitalization.

Interestingly, the hypercholesterolemia was found to be positively associated with the prognosis, in particular favorable functional outcomes, whereas the preexisting of statin use showed no association with the mortality and disability in patients with SICH. Similar findings were found in a large cohort study that showed low cholesterol level was associated with poor functional and death at 3 months after event (Chen et al., 2017).

Our study has a limitation that it does not include long‐term follow‐up. Despite all efforts, the prognosis of SICH is still devastating and is associated with poor outcomes compared to an ischemic stroke.

In summary, SICH is a lethal disease apart from the management and maximal care. The survival is related to factors that are in whole or in part uncontrollable.

## DISCLOSURE

The data were presented in the European Stroke Organization Conference 2018. The abstract was published in the European Stroke Journal 2018.

## CONFLICT OF INTEREST

On behalf of all authors, the corresponding author declares that no potential conflict of interest with respect to the research, authorship, and/or publication of this article is present.

## Data Availability

The data that support the findings of this study are available from the corresponding author upon reasonable request.
